# Diffusion Tensor Imaging, Structural Connectivity, and Schizophrenia

**DOI:** 10.1155/2011/709523

**Published:** 2011-07-17

**Authors:** Thomas J. Whitford, Marek Kubicki, Martha E. Shenton

**Affiliations:** ^1^Psychiatry Neuroimaging Laboratory, Department of Psychiatry, Brigham and Women's Hospital, Harvard Medical School, Boston, MA 02215, USA; ^2^Melbourne Neuropsychiatry Centre, Department of Psychiatry, The University of Melbourne and Melbourne Health, Level 3, Alan Gilbert Building, 161 Barry Street, Carlton South, Melbourne, VIC 3053, Australia; ^3^Clinical Neuroscience Division, Laboratory of Neuroscience, Department of Psychiatry, VA Boston Healthcare System and Harvard Medical School, Brockton, MA 02301, USA; ^4^Surgical Planning Laboratory, MRI Division, Department of Radiology, Brigham and Women's Hospital, Harvard Medical School, Boston, MA 02155, USA

## Abstract

A fundamental tenet of the “disconnectivity” theories of schizophrenia is that the disorder is ultimately caused by abnormal communication between spatially disparate brain structures. Given that the white matter fasciculi represent the primary infrastructure for long distance communication in the brain, abnormalities in these fiber bundles have been implicated in the etiology of schizophrenia. Diffusion tensor imaging (DTI) is a magnetic resonance imaging (MRI) technique that enables the visualization of white matter macrostructure *in vivo*, and which has provided unprecedented insight into the existence and nature of white matter abnormalities in schizophrenia. The paper begins with an overview of DTI and more commonly used diffusion metrics and moves on to a brief review of the schizophrenia literature. The functional implications of white matter abnormalities are considered, particularly with respect to myelin's role in modulating the transmission velocity of neural discharges. The paper concludes with a speculative hypothesis about the relationship between gray and white matter abnormalities associated with schizophrenia.

## 1. Introduction

A notable feature of the neuroimaging literature in schizophrenia is the sheer number of brain structures that have been implicated as abnormal in patients with this disorder. The temporal cortices, medial temporal structures, and frontal lobes have consistently been shown to be morphologically and cytoarchitecturally abnormal in patients with schizophrenia, and abnormalities in the parietal and cingulate cortices, basal ganglia, and cerebellum are also common [1]. However, despite the multitude of gray matter (GM) structures that have been reported to be structurally compromised in patients with schizophrenia, it is notable that there are still no reliable biomarkers for the disease—in contrast, for example, to the amyloid plaques and neurofibrillary tangles that are prognostic for Alzheimer's disease. Furthermore, it is also notable that while GM abnormalities are ubiquitous in schizophrenia, there is no GM structure that is universally atypical in patients with the disease, or to which damage has consistently been shown to trigger psychotic symptoms [2].

Possibly in response to the ongoing failure to identify a distinctive neuropathological signature for the disorder, there has been a movement away from the idea that schizophrenia is caused by a focal neural insult in favor of the idea that schizophrenia arises from abnormal neural communication. The fundamental tenet of the “disconnectivity” theories of schizophrenia is that rather than being caused by normal interactions between pathologic GM structures, the symptoms of schizophrenia instead arise from pathologic interactions between pathologic GM structures [3]. For example, Frith [4] has suggested that delusions of control could arise because of disrupted communication between frontal areas initiating movement and parietal areas processing the sensory consequences of that movement. Crow [5], on the other hand, has suggested that auditory hallucinations could arise from aberrant communication between language-related regions in the temporal cortices, bilaterally. Furthermore, while some “disconnectivity” theories have emphasized the role of aberrant synaptic plasticity in the etiology of schizophrenia [6, 7], others have focused on the role of structural abnormalities in the fibers connecting spatially disparate populations of neurons—that is, the white matter (WM). 

WM is primarily constituted of the phospholipid processes of oligodendrocytes, which are a class of neuroglia. These processes, which are known as myelin, wrap around axons in the central nervous system, insulating the membrane and increasing the conduction velocity of action potentials. Myelinated axons with similar destinations bundle together into fiber tracts, and it is these fiber tracts that constitute the infrastructure for long-distance communication between spatially disparate GM regions. In light of the fact that fiber tract damage can disrupt or even disable communication between connected brain regions [8], and given that psychotic symptoms are unusually common in patients with WM diseases (such as metachromatic leukodystrophy [9] and multiple sclerosis [10]), a number of researchers have speculated as to the existence and implications of WM abnormalities in patients with schizophrenia, [2, 11–14]. The results of genetic analyses that have revealed the abnormal expression of several myelination-related genes in patients with schizophrenia [15] have also contributed to an increased interest into the role of WM abnormalities in the disorder. 

**Figure 1:**
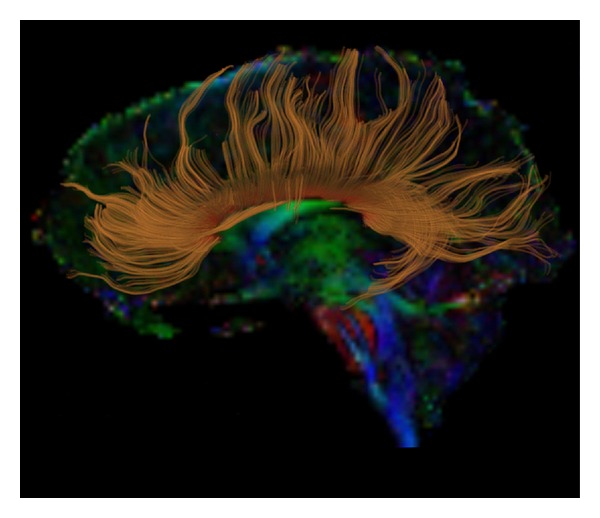
Fiber tractography of the corpus callosum (in gold) overlaid onto a color-by-orientation image extracted from a Diffusion-Tensor Image.

While the basic idea that WM abnormalities could underlie the symptoms of schizophrenia is not new (Bleuler discussed the possibility in 1911), it has only recently become the topic of focused empirical investigation. A major reason for this is technical: WM is notoriously difficult to measure *in vivo *via conventional structural brain imaging techniques such as magnetic resonance imaging (MRI) because of its apparent homogeneity. Furthermore, imaging WM with functional imaging techniques such as positron emission tomography (PET) and functional MRI (fMRI) is also problematic, due to the fact that the metabolic profiles of oligodendrocytes are at least partially independent of task-induced changes in neuron metabolism. And while several *in vitro *stereologic studies have been undertaken in schizophrenia patients postmortem, an acknowledged disadvantage of the technique is the generally suboptimal quality of the tissue, unless a postmortem can be arranged quickly [16]. Given these methodological limitations, it is fair to say that the development of diffusion tensor imaging (DTI) in the 1980s [17] opened the door to the direct empirical investigation of WM abnormalities in patients with schizophrenia, by enabling an accurate quantification of WM integrity *in vivo*. An example of how DTI can be used to identify specific WM fiber bundles (in this case, the corpus callosum), based on their diffusion properties, is displayed in Figure 1. 

## 2. Diffusion Tensor Imaging—A Brief Introduction

**Figure 2:**
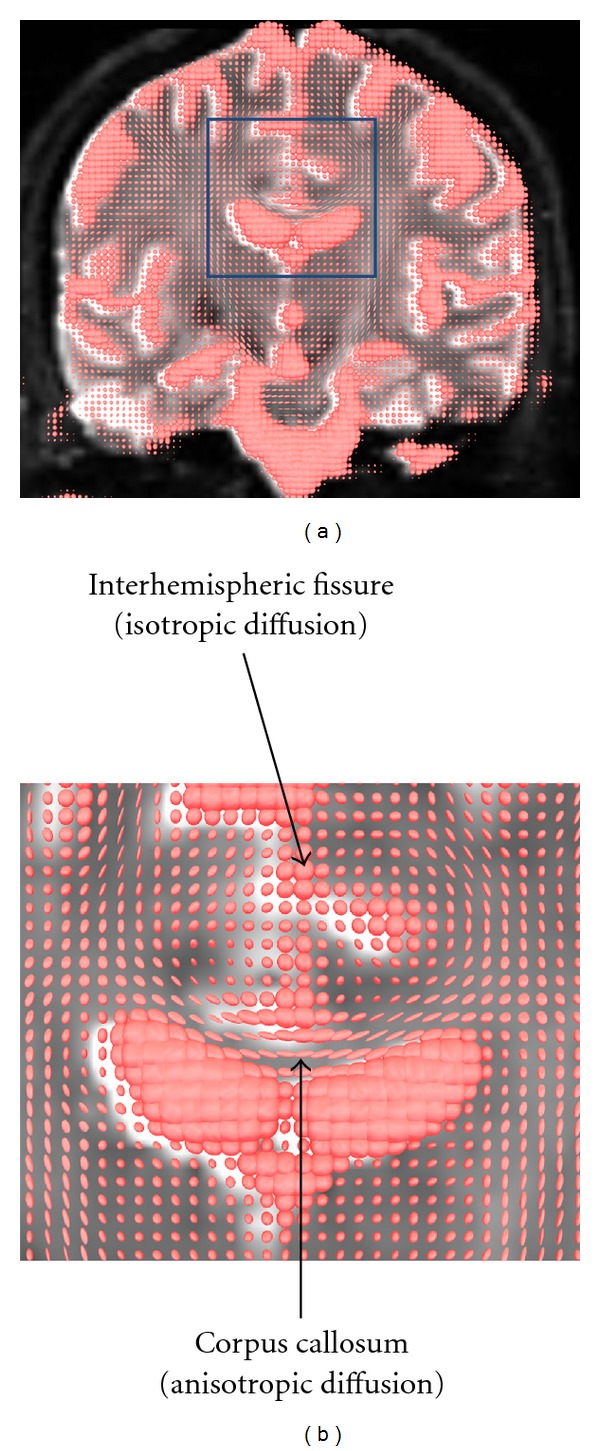
The shape of water diffusion in the different tissues of the brain. (a) depicts the variations in the shape of water diffusion across a coronal slice of brain tissue, while (b) is a zoomed-in section of (a). As illustrated in (b), the shape of diffusion in the inter-hemispheric fissure is approximately spherical, as there are few obstacles to diffusion in the CSF. In contrast, the tightly aligned, myelinated fibers of the corpus callosum form a significant obstacle to diffusion. Diffusion is more likely to occur parallel to the white matter fibers as opposed to perpendicular to them, and thus the shape of diffusion is ellipsoidal in these voxels.

DTI enables inferences to be made in terms of the integrity and orientation of fiber tracts on the basis of patterns of water diffusion. DTI is noteworthy in that it can provide information in terms of WM anatomy that is simply not accessible with any other method—either *in vivo *or *in vitro*. Diffusion—otherwise known as Brownian motion—refers to the random movement of particles, such as water molecules, as a result of unpredictable, thermally driven molecular collisions. In an unrestricted medium, a water molecule is equally likely to move in one direction as another. However, in brain tissue the diffusion of water molecules is restricted by obstacles in the local environment such as cell membranes, myelin sheaths, and macromolecules. The extent to which diffusion is restricted differs between the different tissues of the brain. In the cerebrospinal fluid (CSF), for example, there are relatively few obstacles to diffusion, and hence the average shape of the resultant diffusion is approximately spherical: this is known as isotropic diffusion. In contrast, in a WM fiber bundle the densely packed and homogeneously oriented bundles of myelinated axons provide a considerable barrier to water diffusion. In this case, water is more likely to diffuse parallel to the WM bundle rather than perpendicular to it, which will make the shape of the resultant diffusion less spherical and more ellipsoidal: this is known as anisotropic diffusion—see Figure 2. By calculating the distance in which water diffuses in a given voxel in a given amount of time (i.e., in the order of milliseconds) for at least six noncollinear directions, it is possible to reconstruct a three-dimensional shape that best describes the pattern of water diffusion occurring in a given voxel. The shape best describing this pattern of diffusion is conventionally modeled as an ellipsoid, and the important point is that the volume and shape of this ellipsoid provide information about the diffusion properties and hence the microstructural features of the brain tissue. 

Three of the more common diffusivity indices that have been examined in the DTI literature are fractional anisotropy (FA), mean diffusivity (MD), and, more recently, Mode. FA is a measure of the sphericity of the diffusion ellipsoid. FA can vary between values of zero and one, with completely spherical diffusion having a value of zero and perfectly aspherical (e.g., linear) diffusion having a value of one. In a WM bundle, reduced FA is generally assumed to reflect damage to the axon membrane, reduced axonal myelination, reduced axonal packing density, and/or reduced axonal coherence [18], while increased FA has been suggested to reflect supranormal levels of myelination or axonal sprouting [19]. On the other hand, MD is dependent on the volume of the diffusion ellipsoid, that is, the average displacement of water molecules as a result of diffusion in a given amount of time. MD is highest in tissues where there are few impediments to water diffusion (e.g., CSF), and lowest in tissues where diffusion is restricted at least one direction (e.g., WM). Although FA and MD are (almost) mathematically independent, they are generally found to be inversely related in the brain, such that tissue showing highly anisotropic diffusion (such as WM) generally shows low MD. Mode is a relatively recently developed index that provides additional information in terms of the 3D shape of the diffusion ellipsoid than that provided by FA. Roughly speaking, for a given FA value, Mode describes whether the diffusion ellipsoid is shaped like a cylinder (i.e., having highly “tubular” anisotropy) or like a disk (i.e., having highly “planar” anisotropy) [20]. When considered in combination with FA, the Mode of a diffusion ellipsoid provides unique information as to the microstructural features of the underlying WM. For example, the presence of fiber crossings has been associated with reductions in Mode [21, 22]. Hence, the finding that schizophrenia patients show supranormal levels of Mode in the corpus callosum has been argued to reflect a reduction in the density of fibers adjacent to this fasciculus [23].

## 3. White Matter Abnormalities in Schizophrenia—Do They Exist and What Do They Mean?

The first DTI study that investigated WM abnormalities in patients with schizophrenia was performed by Buchsbaum et al. [24] in 1998. Since that time, over 100 DTI studies have been performed on patients with schizophrenia. By far the most consistent finding has been of FA reductions in the patients, with significant reductions being reported in the corpus callosum, superior longitudinal fasciculus, inferior longitudinal fasciculus, arcuate fasciculus, uncinate fasciculus, cingulum bundle, and fornix [13, 18, 23, 25–47]. The observation of FA reductions in patients with schizophrenia raises the question as to what are the physiologic underpinnings of these FA abnormalities. Research by Beaulieu [48] indicates that axonal membranes are the primary determinant of anisotropic water diffusion in fiber bundles, with axonal myelination also modulating anisotropy to a significant, albeit lesser, extent. Hence one possibility is that the reduced FA observed in schizophrenia patients is due to a pathologic reduction in the number of neurons, and hence their associated axons. However, there is little evidence to suggest a significant reduction in neuron number in the brains of schizophrenia patients relative to healthy people [49]. By contrast, there is evidence to suggest that the characteristic GM reductions observed in schizophrenia patients are likely the result of a reduction in the density of dendrites, spines, axon-terminals, and neuroglia [50]. In light of this, an alternative possibility is that the FA abnormalities in schizophrenia are caused by damage to the myelin-sheaths of oligodendrocytes.

It has been demonstrated that myelin abnormalities alone can result in significant reductions in FA. For example, Nair and colleagues [51] compared wild-type mice and transgenic *shiverer* mice (who show abnormalities in myelin but not to the axon membrane) and found that the *shiverer* mice had significantly decreased FA in the corpus callosum. Furthermore, Song and colleagues [52] found evidence indicating that it is possible to distinguish between axonal damage and dysmyelination on the basis of the distinctive patterns of diffusivity abnormalities induced by these injuries. Specifically, Song et al. [52] demonstrated that while the amount of diffusion perpendicular to the principal orientation of the optic nerve (which they termed “radial diffusivity”) was increased in *shiverer* mice with severe dysmyelination, the amount of diffusion parallel to the tract (“axial diffusivity”) was unaffected. Conversely, Song et al. [53] demonstrated that axonal injury concurrent with myelin preservation resulted in a decrease in axial diffusivity but no change in radial diffusivity. The significance of these findings is clear in the context of a recent study by Ashtari et al. [54] who found that schizophrenia patients exhibited abnormally increased radial diffusivity in the inferior longitudinal fasciculus, but no difference in axial diffusivity, putatively indicating the presence of myelin abnormalities but the absence of axonal abnormalities. And finally, in an electron microscopy study (with a very short postmortem delay of four to six hours), Uranova et al. [55] reported evidence of oligodendrocyte degeneration and myelin damage in the prefrontal cortex and caudate nucleus of patients with schizophrenia. When taken in combination, these results suggest that myelin abnormalities are at least partially responsible for the FA reduction typically observed in patients with schizophrenia. If myelin abnormalities are at least partially responsible for the FA reductions in patients with schizophrenia, then what causes these abnormalities? Furthermore, what is the relationship (if any) between the myelin abnormalities inferred with DTI and the GM abnormalities that have been consistently observed in these patients both *in vivo* and *in vitro*? 

It is well established that one of the principal functions of myelin is to increase the transmission velocity of action potentials traveling along axons by increasing membrane capacitance and reducing ion leakage through the axon membrane [8]. Furthermore, there is evidence to suggest that diffusivity indices are correlated with indices of conduction velocity measured via electroencephalography. For example, Whitford and colleagues [56] found a significant negative correlation between the FA of the visual fibers of the corpus callosum and interhemispheric transfer time as measured with visually evoked potentials in both schizophrenia patients and healthy controls. Given the evidence indicating that patients with schizophrenia exhibit myelin abnormalities in at least some WM bundles, particularly in those bundles that connect the frontal lobe with the rest of the brain, it seems reasonable to assume that this would result in significant transmission delays in neural communication between spatially disparate GM regions, such as between the frontal lobe and the temporal cortex (see [57]). And as has been pointed out by both Fields [11] and Aboitiz [58] even small transmission delays resulting from damaged myelin could severely disrupt the synchronicity of disparate GM regions. 

In light of this, it is notable that several studies have reported evidence of abnormal synchrony in the electroencephalogram (EEG) in patients with schizophrenia [59, 60]. This finding is particularly salient given the role that neural synchrony has been proposed to play in perceptual (and possibly cognitive) integration—abilities that are ubiquitously aberrant in patients with schizophrenia. Recent work into the functional role of the N-methyl D-aspartate- (NMDA-) receptor has also led to increased interest in terms of the role of neural synchronization in the etiology of schizophrenia. The NMDA receptor is distinctive in that it is both ligand and voltage-gated [61]. That is, the receptor will only allow cation influx if glutamate is bound to the receptor at the same time as the postsynaptic membrane is depolarized. Given its distinct physiology, the NMDA-receptor has been implicated as a “synchrony detector” that may play a role in determining which synapses are eliminated during development [62]. More specifically, synchronous synaptic activity (which has been associated with NMDA-receptor activation [63]) has been suggested to result in synaptic preservation, while asynchronous activity has been suggested to facilitate or even promote synaptic pruning [64]. 

Taken together, these findings might be explained by a speculative hypothesis as to the relationship between the GM and WM abnormalities present in patients with schizophrenia. The hypothesis, which has been described in detail elsewhere [65], suggests that some cases of schizophrenia arise because of currently undetermined developmental/environmental triggers causing the abnormal expression of myelin-related genes during the normative peri-pubescent period of myelination of the association cortices. The resultant myelin is consequently structurally damaged and hence functionally damaged in its ability to insulate the axon membranes and increase conduction velocity. This results in small but significant transmission delays in communications between spatially disparate GM structures. These transmission delays result in a disruption in neural synchronization, such as between primary neural discharges and their corollaries [57]. This results in both the symptoms of psychosis and the exaggerated synaptic pruning (possibly mediated by the NMDA-receptor) in the GM regions connected by this functionally retarded WM. In a similar vein, Moises et al. [66] have suggested that there may be a causal relationship between abnormalities in glial growth factors and synaptic destabilization in patients with schizophrenia.

This theory is clearly speculative and should be treated with caution in the absence of a great deal more empirical evidence. However, if nothing else, this theory acts as an example of how WM abnormalities could potentially underpin both the symptoms of schizophrenia and the GM atrophy characteristic of the disease. Moreover, if the WM abnormalities exhibited by schizophrenia patients did indeed underlie their psychological and physiological pathologies, then this could provide an alternative target for pharmacologic interventions. For example, if myelin abnormalities were ultimately found to underlie the hyperdopaminergic state characteristic of schizophrenia, which the results of Roy [67] at least tentatively suggest, then targeting the root of the problem with medications used to preserve WM (such as those in development for the treatment of multiple sclerosis [68]) would potentially provide a useful adjunct or even a better alternative to the dopamine antagonists currently used in antipsychotic pharmacotherapy. It has also been suggested that the efficacy of current antipsychotic medications may, at least in part, be due to their effects on periadolescent myelination [69]. The idea that pharmacotherapy could be used to target the root cause of the abnormal neurochemical profile exhibited by patients with schizophrenia is a tantalizing prospect, and one that is worthy of pursuit in the years ahead.

## 4. Conclusions

DTI is unique in its ability to investigate *in vivo *the structural integrity of WM fiber bundles, which represent the neuroanatomical infrastructure for long-distance communication in the brain. The consistency of WM abnormalities observed in schizophrenia patients via DTI has been one of the factors underlying the emergent (or rather, re-emergent) conceptualization of schizophrenia as a disorder of abnormal connectivity between spatially disparate brain structures. This conceptualization provides a novel theoretical framework in terms of the etiology of the disease and opens the door for the development of novel diagnostic tools and novel treatment. When considered in combination with other biomedical tools tuned to different aspects of brain physiology (e.g., oxygenation with fMRI, neurochemistry with nuclear magnetic resonance spectroscopy, electrodynamics via EEG, genetic profile via microarray technology, etc.), the development of DTI as a WM-imaging tool provides us with an unprecedented opportunity to understand the root causes of schizophrenia—an understanding that is necessary for the targeted development of successful treatment programs. The promise of new, knowledge-based breakthroughs in the treatment of this devastating disorder makes this an exciting time to be involved in schizophrenia research.
